# The protective effects of XH‐105 against radiation‐induced intestinal injury

**DOI:** 10.1111/jcmm.14159

**Published:** 2019-01-20

**Authors:** Ying Cheng, Yinping Dong, Qinlian Hou, Jing Wu, Wei Zhang, Hongqi Tian, Deguan Li

**Affiliations:** ^1^ Tianjin Key Laboratory of Radiation Medicine and Molecular Nuclear Medicine Institute of Radiation Medicine, Chinese Academy of Medical Science & Peking Union Medical College Tianjin China; ^2^ Center for Marine Bioproducts Development College of Medicine and Public Health, Flinders University Bedford Park, Adelaide South Australia Australia

**Keywords:** apoptosis, DNA damage, p53, small intestine, total body irradiation

## Abstract

Radiation‐induced intestinal injury is one of the major side effects in patients receiving radiation therapy. There is no specific treatment for radiation enteritis in the clinic. We designed and synthesized a new compound named XH‐105, which is expected to cleave into polyphenol and aminothiol in vivo to mitigate radiation injury. In the following study, we describe the beneficial effects of XH‐105 against radiation‐induced intestinal injury. C57BL/6J mice were treated by gavage with XH‐105 1 hour before total body irradiation (TBI), and the survival rate was monitored. Histological changes were examined, and survival of Lgr5^+^ intestinal stem cells Ki67^+^ cells, villi^+^ enterocytes and lysozymes was determined by immunohistochemistry. DNA damage and cellular apoptosis in intestinal tissue were also evaluated. Compared to vehicle‐treated mice after TBI, XH‐105 treatment significantly enhanced the survival rate, attenuated structural damage of the small intestine, decreased the apoptotic rate, reduced DNA damage, maintained cell regeneration and promoted crypt proliferation and differentiation. XH‐105 also reduced the expression of Bax and p53 in the small intestine. These data suggest that XH‐105 is beneficial for the protection of radiation‐induced intestinal injury by inhibiting the p53‐dependent apoptosis signalling pathway.

## 1 INTRODUCTION

The small intestine is one of the most sensitive organs for ionizing radiation. The main symptoms of radiation‐induced intestinal damage include anorexia, vomiting, diarrhea, dehydration, systemic infection, and in extreme cases, septic shock and death.^1^ Radiation‐induced intestinal damage seriously affects the treatment of patients with abdominal or pelvic tumours, reducing the quality of life of patients. However, there is no specific treatment for radiation enteritis in the clinic. Therefore, the development of efficient radiological intestinal damage protectors is an important area in radiation protection.

Natural anti‐oxidation agents exist widely in herbs and fruits and mainly include flavonoids and polyenes that could be used as radioprotectors.^2^, ^3^, ^4^, ^5^ Both of these agents have advantages of low toxicity and moderate efficacy, but display poor stability and bioavailability.^6^ Aminothiols emerge as the most promising compounds, especially after amifostine was discovered and approved by the Food and Drug Administration. Although amifostine is currently used clinically, its drug toxicity, limited times of protection and unfavourable routes of administration^7^ limit the utility of the drug in non‐clinical settings. The most probable protective mechanisms of aminothiols are that the aminothiol radioprotectors donate a H atom, scavenge hydroxyl radical or other reactive oxygen species (ROS), chemically repair DNA radicals and deplete oxygen.^8^, ^9^

In this study, we examined the natural anti‐oxidation agent quercetin, which belongs to flavonoids family derived from plants, as a lead compound and modified the molecular structure. At the same time, we also tried to combine the aminothiol analogue and the natural anti‐oxidation agent together with different linkers to retain the efficacy of aminothiol and the safety property of natural anti‐oxidation agents respectively. Furthermore, the two functional fragments modulated the pharmacokinetic profile for each other, and they were mutually prodrug to each other. The new compound was expected to cleave into polyphenol and aminothiol in vivo to mitigate radiation injury by their ROS scavenger capabilities. Based on this concept, we designed and synthesized the compound XH‐105.

To define the effects of XH‐105 on intestinal radiation injury, we used a mouse model with exposure to 9.0 Gy total body irradiation (TBI). We found that XH‐105 could improve the survival rate of mice and intestinal epithelium cells (IECs). The crypt‐villous structure injuries of small intestinal and the apoptosis of IECs induced by TBI were mitigated by XH‐105.

## 2 MATERIALS AND METHODS

### 2.1 Synthesis of 2,2‐dimethylthiazolidine‐3‐carbonyl chloride

To a solution of bis(trichloromethyl) carbonate (4.05 g, 13.66 mmol) (Energy Chemical, Shanghai, China) in anhydrous tetrahydrofuran (30 mL), 2,2‐dimethylthiazolidine (4 g, 44.87 mmol) (Energy Chemical) was added in parts and triethylamine (8.1 mL) was added dropwise; the mixture was stirred under nitrogen atmosphere and in an ice water bath for 1 hour and then at room temperature for 10 hours. The reaction mixture was filtered, and the residue was washed three times with dichloromethane (20 mL). The filtrate phase was combined and evaporated in vacuo. The resultant residue was used directly for the next step.

### 2.2 Synthesis of 2‐(3,4‐bis((2,2‐dimethylthiazolidine‐3‐carbonyl)oxy)phenyl)‐4‐oxo‐4H –chromene‐3,5,7‐triyl tris(2,2‐dimethylthiazolidine‐3‐carboxylate)

Triethylamine (6 mL), 4‐dimethylaminopyridine (157 mg, 1.29 mmol) and 2,2‐dimethylthiazolidine‐3‐carbonyl chloride were added to a solution of 2‐(3,4‐dihydroxyphenyl)‐3,5,7‐trihydroxy‐4H‐chromen‐4‐one (1.3 g, 4.30 mmol) (SHUYA Chemical Science and Technology, China) in N,N‐dimethylformamide (30 mL),and the mixture was stirred at 0°C for 1 hour and at room temperature for 10 hours. The reaction mixture was poured into a mixture solvent (500 mL, v/v/v, MeOH:H_2_O:dimethyl sulfoxide [DMSO] = 8:1.5:0.5), and then water was added dropwise until the product precipitated. The residue was then filtered and washed three times with water (20 mL).The filter cake was then dried in vacuo and produced the title compound as a yellow solid (3.35 g, 65%). Liquid chromatograph mass spectrometer (LC‐MS) was carried out on a Waters 3100 Mass Detector with an Agilent ZORBAX column (C18, 2.1 × 50 mm, 3.5 μm). ^1^H NMR spectra were obtained using a Bruker spectrometer at 400 MHz. LC‐MS: R_T_ = 7.06 minutes, [M + H]^+^ = 1018.32, Calculated: 1018.25. ^1^H NMR (400 MHz, DMSO‐d6) δ 7.85 (d, J = 15.7 Hz, 2H), 7.64 (s, 1H), 7.56 (d, J = 8.4 Hz, 1H), 7.18 (s, 1H), 4.02 (d, J = 26.2 Hz, 10H), 3.15 (d, J = 30.1 Hz, 10H), 1.77 (d, J = 17.1 Hz, 30H).

### 2.3 Animals

Male C57BL/6 mice (8‐10 weeks) were purchased from Beijing HFK Bioscience Co., Ltd. (Beijing, China). Animals were bred in the certified animal facility at the Institute of Radiation Medicine (IRM) of the Chinese Academy of Medical Sciences (CAMS).

### 2.4 Ethics approval and consent to participate

All experimental procedures were carried out in accordance with the NIH Guidelines for the Care and Use of Laboratory Animals and were approved by the Institutional Animal Care and Use Committee of the IRM, CAMS (Permit Number 2017053). The animals were cared for in accordance with the guidelines of the National Animal Welfare Law of China.

### 2.5 Irradiation and treatment

Mice were exposed to ionizing radiation by using a ^137^Cs source following an Exposure Instrument Gammacell‐40 (Atomic Energy of Canada Lim, Chalk River, ON, Canada) at a dose rate of 1.0 Gy/min. The mice were exposed to 7.5, 9.0 and 11.0 Gy TBI in the survival experiments (n = 10).

In the remaining experiments, animals were divided randomly into three groups (n = 5): (a) control; (b) IR + vehicle; (c) IR + XH‐105 and received 9.0 Gy TBI. XH‐105 was dissolved in 4% DMSO, 96% of PEG 400 was added after heating for a final concentration of 10 mg/mL. Individual mice in the IR + XH‐105 group received a dose of 100 mg/kg XH‐105 administered by gavage 1 hour before irradiation. Mice in the Control and IR groups were treated with vehicle similarly to the procedure described for the XH‐105 treatment.

### 2.6 Histological analysis

Three days after IR, mice were killed, and the small intestines were collected, stained with haematoxylin‐eosin (H&E) and analysed under a microscope. For morphological analysis, six circular transverse sections were analysed per mouse in a blind manner from coded digital H&E‐stained photographs to measure the villi length and crypt number by using ImageJ 1.37 software.

### 2.7 Immunohistochemistry analysis

The 4‐μm‐thick sections of paraffin‐embedded small intestine sections were dewaxed and rehydrated with citrate buffer. Then, the sections were boiled in 10 mM/L citrate buffer solution (pH 9.0) for antigen retrieval according to standard procedures. After antigen retrieval, the sections were incubated with serum for 1 hour at room temperature to block non‐specific antigen‐binding sites; then, the sections were incubated with anti‐Lgr5 antibody (1:50 dilution; Abcam, Cambridge, MA), anti‐Ki67 antibody (1:300 dilution; Novus, Littleton, CO), anti‐lysozyme (1:800 dilution; Abcam) or anti‐villi (1:800 dilution; Abcam) overnight at 4°C. Sections were then incubated in secondary antibody for 30 minutes at 37°C. Positive cells were detected using a DAB kit (Sigma Aldrich, St. Louis, MO). The images were captured, and positive staining was quantified objectively by the integrated performance primitives (IPP) software as described previously in a blinded fashion.

### 2.8 TUNEL assay

The 3‐μm‐thick sections were treated according to the manufacturer's protocols (Roche, Mannheim, Germany). Sections were analysed by light microscopy.

### 2.9 Isolation of intestinal crypt cells

The method of isolating intestinal crypts was described.^10^, ^11^ Briefly, after flushing with ice‐cold PBS, the small intestines were chopped into small pieces and then placed into cold crypt chelating buffer for 30 minutes. After rinsing twice with ice‐cold PBS, the fragments were resuspended in cold dissociation buffer. The solution was filtered through a 70 μm strainer to remove the villus fraction and to collect the crypt fraction. The crypt fraction was centrifuged to isolate the single cells.

### 2.10 Western blot analysis

Protein was extracted from small intestinal crypt cells with ice‐cold lysis buffer (Solarbio Science and Technology, Beijing, China). The protein concentration was quantified using the bicinchoninic acid (BCA) Protein Assay Kit (Beyotime, Shanghai, China), and equal amounts of protein were resolved by a SDS‐PAGE gel. The blocked membrane was incubated using antibodies against anti‐Bax (1:1000 dilution; Ruiyingbio, Suzhou, China) or against β‐tubulin (1:2500 dilution; Proteintech, Wuhan, China) overnight at 4°C. Then, the membranes were incubated with a suitable horseradish peroxide‐conjugated secondary antibody for 1‐2 hours at room temperature. Finally, the chemiluminescent substrate is used to detect protein.

### 2.11 Immunofluorescence analysis

The paraffin‐embedded sections of the small intestine were subjected to antigen retrieval as described above and then washed thoroughly with PBS. The sections were blocked with 5% goat serum for 30 minutes at room temperature and incubated with anti‐caspase‐8 (1:100 dilution; CST, MA, USA), anti‐caspase‐9 (1:1000 dilution; CST), anti‐γH2AX (1:1000 dilution; BD biosciences, NJ, USA) or anti‐p53 (1:1000 dilution; Ruiyingbio) overnight at 4°C. After washing with PBS, sections were incubated in the secondary antibody for 40 minutes at 37°C while avoiding light. Sections were finally sealed with 4',6‐diamidino‐2‐phenylindole‐containing sealing agent. The images were captured by laser scanning confocal microscopy.

### 2.12 Statistical analysis

Mice survival curves were analysed by the Kaplan‐Meier method using GraphPad Prism 6.0 software for Mac. The data were expressed as the mean ± SD. ANOVA test was used to analyse differences among groups, and Student's *t* test was used to analyse the difference between two groups.

## 3 RESULTS

### 3.1 Synthesis and characterization of XH‐105

**Figure 1:**
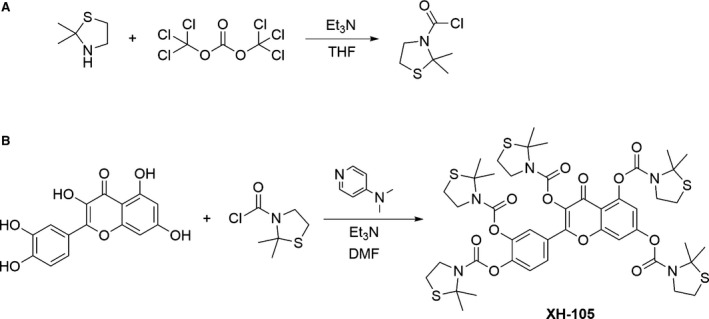
Synthesis Route and Molecular Structure of XH‐105. A, The 2,2‐dimethylthiazolidine was reacted with bis(trichloromethyl) carbonate with triethylamine as base to prepare thiazolidine‐3‐carbonyl chloride. B, Thiazolidine‐3‐carbonyl chloride was coupled with Quercetin in the presence of triethylamine and 4‐dimethylaminopyridine to afford the product XH‐105

Based on the design concept, we designed and prepared XH‐105, of which the molecular structure and synthetic routes are shown in Figure 1. The synthetic procedures are depicted in the Supplementary Materials. Briefly, 2,2‐dimethylthiazolidine was reacted with bis(trichloromethyl) carbonate with triethylamine as the base to prepare 2,2‐dimethylthiazolidine‐3‐carbonyl chloride. Then, the as‐prepared intermediate was further coupled with quercetin molecules in the presence of triethylamine and 4‐dimethylaminopyridine to afford the 2‐(3,4‐bis((2,2‐dimethylthiazolidine‐3‐carbonyl)oxy) phenyl)‐4‐oxo‐4H‐chromene‐3,5,7‐triyl tris(2,2‐dimethylthiazolidine‐3‐carboxylate) as a product with the isolation yield of 65%. The structure was characterized by NMR and ESI‐MS. The ^1^H‐NMR and ESI‐MS spectra of XH‐105 are shown in Figure S1A and Figure S1B respectively. The results indicated that the new compound XH‐105 was successfully prepared with the facile synthetic approach.

### 3.2 XH‐105 improves the survival rate of mice after TBI

**Figure 2:**
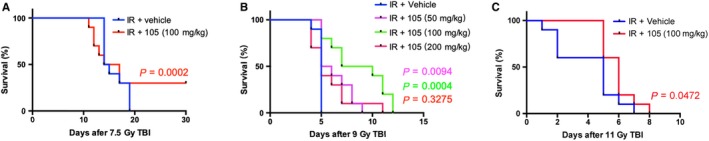
XH‐105 improves the survival of mice after 9.0 Gy total body irradiation (TBI). Kaplan‐Meier survival analysis of mice exposed to 7.5, 9.0 or 11.0 Gy TBI. A, 100 mg/kg XH‐105 treated mice have 30% survival beyond 30 d post 7.5 Gy TBI, compared with IR mice with 100% mortality within 20 d of radiation exposure (*P* < 0.05, n = 10 per group). B, three doses (50, 100, 200 mg/kg) of XH‐105 treated mice show reduced mortality following lethal doses of TBI (9.0 Gy) within 13 d, compare with IR group 100% mortality within 5 d (*P* < 0.05, n = 10 per group). C, The Kaplan‐Meier survival curve of vehicle and 100 mg/kg XH‐105 treated mice (*P* < 0.05, n = 10 per group) after 11.0 Gy TBI. The data were expressed as the percent of surviving mice

To assess the protective effect of XH‐105 on TBI‐induced lethality in mice, we first observed the survival rates of mice after 7.5 Gy TBI (Figure 2A). The 7.5 Gy TBI had a 100% mortality in the vehicle‐treated group within 20 days compared with the 100 m/kg XH‐105‐treated group that had a 30% survival rate beyond 30 days. We treated the mice with three doses of XH‐105 (50, 100 and 200 mg/kg), and then the mice were exposed to 9.0 Gy TBI (Figure 2B). All doses improved the mice survival rate compared to that of the vehicle‐treated group, and in particular, the 100 mg/kg dose dramatically improved the median survival. There was 80% mortality in vehicle‐treated mice at 6 days after 11.0 Gy TBI (Figure 2C), while 60% of mice survived in the 100 m/kg XH‐105‐treated group, suggesting that XH‐105 may have a protective effect on radiation‐induced intestinal injuries in mice. These results indicated that XH‐105 effectively mitigates the TBI‐induced lethality in mice.

### 3.3 XH‐105 reduces the damages of intestinal morphology after TBI

**Figure 3:**
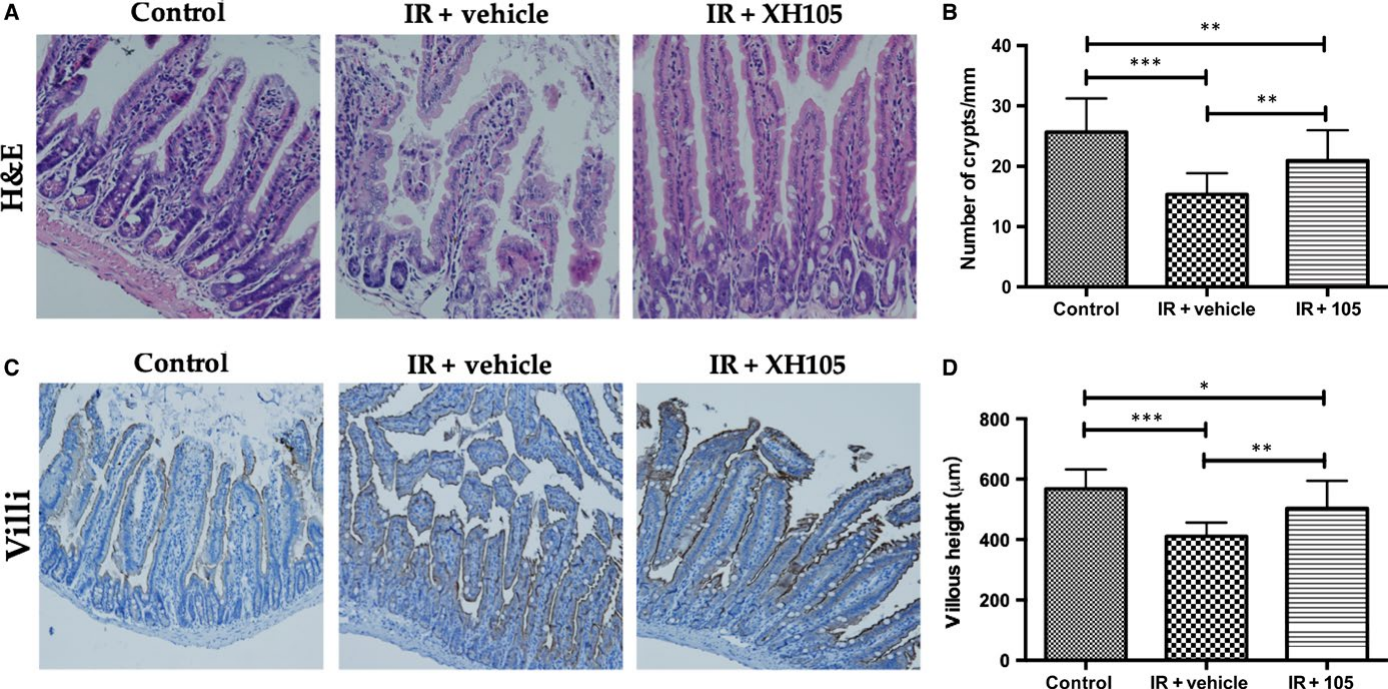
XH‐105 protects the intestinal morphology of mice after 9.0 Gy total body irradiation (TBI). A, Representative images showing the structure in cross‐sections of the small intestine with haematoxylin‐eosin (H&E) stained. B, Histogram showing the number of crypts. C, Immunohistochemistry images showing the expression of Villi. D, Histogram demonstrating villus length in intestinal section from the control group, vehicle‐treated group and XH‐105‐treated group. The results are represented as mean ± SEM, n = 5 mice per group. **P < 0.01, *P < 0.05. Scale bar: 100um and 50um

To determine the effect of XH‐105 on radiation‐induced intestinal injuries, the morphological changes of the small intestine in mice were evaluated. At 3.5 days after 9.0 Gy TBI, irradiated mice treated with vehicle showed significantly shorter villous length (*P* < 0.005) and fewer crypts (*P* < 0.005) than the control group (Figure 3A,B). In comparison to vehicle‐treated mice, XH‐105‐treated mice showed more survival crypts (*P* < 0.01) and an increased villous height (*P* < 0.01). The expression of villi^+^ enterocytes was also affected by radiation (Figure 3C,D), and mice treated with XH‐105 exhibited a significant increase in expression compared with vehicle‐treated mice. These results indicated that XH‐105 treatment can prevent post‐radiation damage of the intestinal villus‐crypt structures in mice.

### 3.4 XH‐105 enhances Lgr5^+^ ISC survival and maintains the regeneration of intestinal cells after TBI

**Figure 4:**
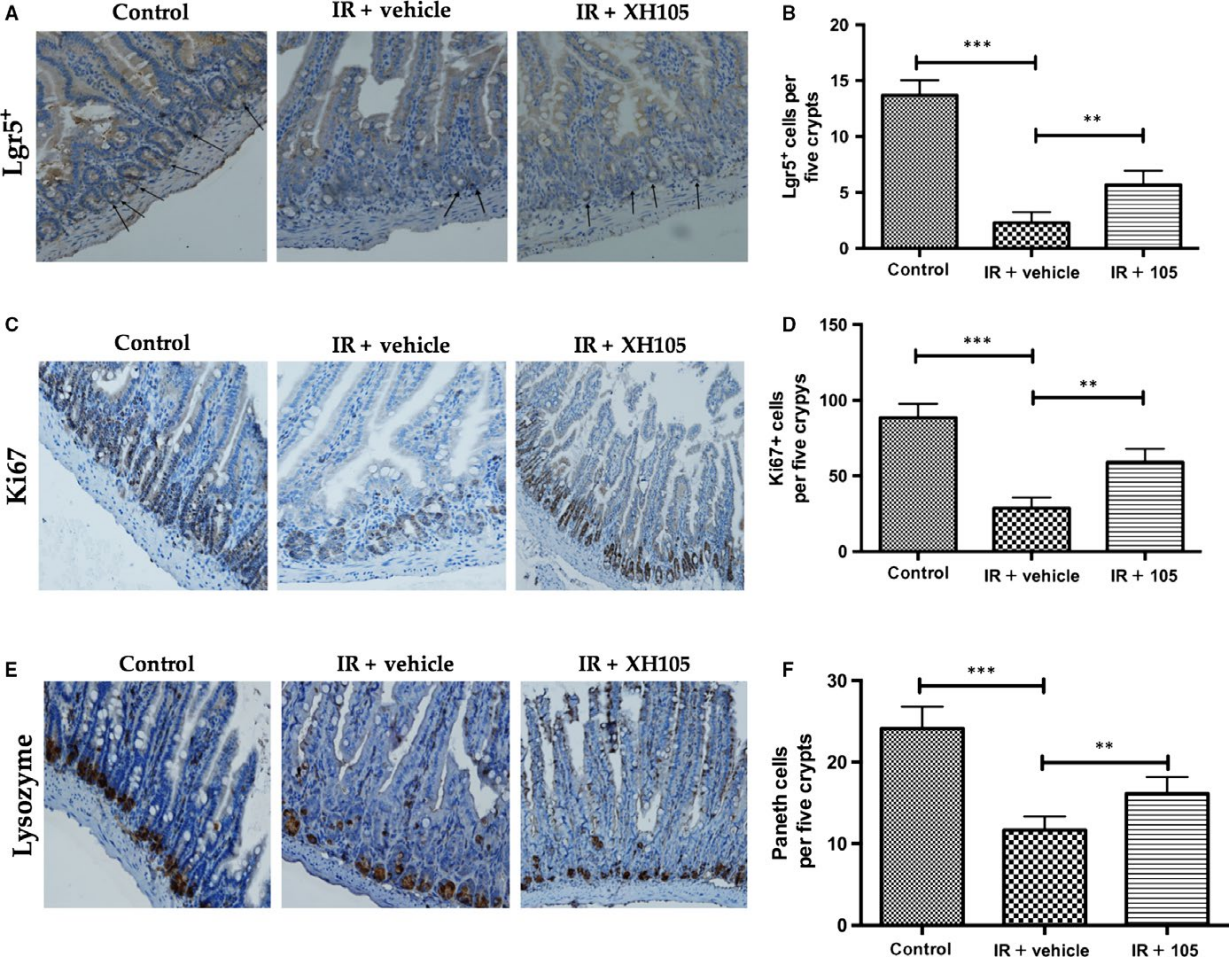
XH‐105 enhances the proliferation and differentiation ability of the Lgr5+ small intestine after 9.0 Gy total body irradiation. The small intestinal sections were analysed by immunohistochemistry. A, Photomicrograph of Lgr5 immunostaining section of control, IR + vehicle and IR + 105 group. B, Histogram showing Lgr5‐positive cells were quantified in five crypts per section. C, Immunostaining images showing Quantitative analysis of Ki67 expression of intestinal crypts. D, Histogram demonstrating Ki67‐positive cells were counted in five crypts per section. E, Representative immunohistochemistry images for lysozyme‐stained sections of small intestine. F, Histogram showing the number of paneth cells per five crypts. The results are represented as mean ± SEM, n = 5 mice per group. **P < 0.01. Scale bar: 50um

To evaluate the effect of XH‐105 on the proliferation and differentiation ability of crypt cells, Lgr5 and Ki67 were identified by immunohistochemistry staining. Lgr5^+ ^intestinal stem cells are indispensable for intestinal regeneration following radiation.^12^ At 3.5 days after 9.0 Gy TBI, the number of Lgr5^+^ intestinal stem cells (ISCs) was significantly increased in the XH‐105‐treated mice than in the vehicle‐treated group (Figure 4A,B). Similarly, the number of Ki67^+ ^positive cells in XH‐105‐treated mice was also markedly greater than those in vehicle‐treated mice after TBI (Figure 4C,D). Paneth cells located at the bottom of intestinal crypts produce lysozymes. We also investigated the changes in Paneth cells 3.5 days after 9.0 Gy TBI (Figure 4F). Similarly, we found that the number of lysozyme^+ ^positive cells (Figure 4E) of XH‐105‐treated group were greater than that of the vehicle‐treated group. In summary, these data clearly indicated that XH‐105 enhances the regenerative response of radiation‐induced intestinal injuries by promoting the differentiation and proliferation of ISCs in the small intestine.

### 3.5 XH‐105 decreases apoptosis of the small intestine after TBI

**Figure 5:**
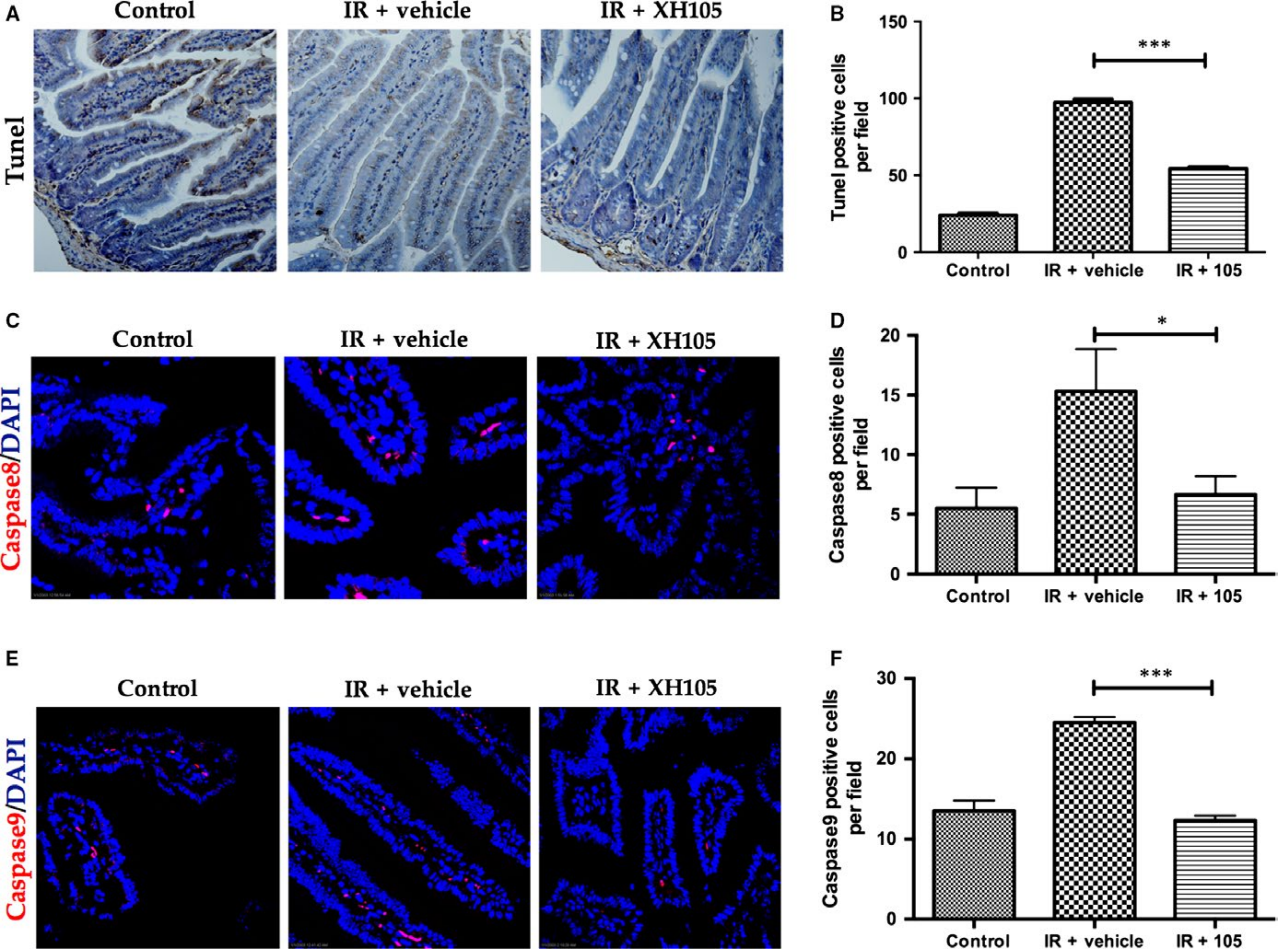
XH‐105 decreases the apoptosis of the small intestine after 9.0 Gy total body irradiation. A, Apoptosis was assayed by terminal deoxynucleotidyl transferase dUTP nick end labeling (TUNEL) staining. B, The number of TUNEL positive cells was quantified per field. The paraffin‐embedded sections of small intestine were analysed by immunofluorescence. C, Representative 4',6‐diamidino‐2‐phenylindole (DAPI) and caspase8‐staining images of the small intestine (red, caspase8; blue, DAPI). D, Caspase8‐positive cells in a single field of view were quantified. E, Photomicrograph of caspase9‐staining images of the small intestine (red, caspase9; blue, DAPI). F, Bar graph showing Quantitative analysis of caspase9‐positive cells per field of view. The results are represented as mean ± SEM, n = 5 mice per group. *P < 0.05, ***P < 0.005. Scale bar: 50um and 10um

To investigate the effect of XH‐105 on apoptosis of the small intestines after IR, we evaluated apoptosis in the small intestine by terminal deoxynucleotidyl transferase dUTP nick end labeling (TUNEL) assay. The results indicated that XH‐105 had a protective role in preventing radiation‐induced intestinal damage by suppressing apoptosis (Figure 5A,B). To further validate our observations, we also analysed the rate of apoptosis by caspase‐8 antibody and caspase‐9 antibody immunofluorescence staining of small intestinal sections from mice after 9.0 Gy TBI. The immunofluorescence staining images showed expression of apoptosis‐related proteins (Figure 5C,E). The mice exposed to radiation increase the number of apoptotic nuclei in the small intestinal crypts of mice. At 3.5 days after 9.0 Gy TBI, XH‐105‐treated mice showed a significantly lower number of apoptotic cells than the vehicle‐treated mice. These data suggested that XH‐105 treatment could decrease the number of apoptotic cells and protect mice from irradiation‐induced intestinal injuries.

### 3.6 XH‐105 attenuates DNA damage of the small intestine after TBI

**Figure 6:**
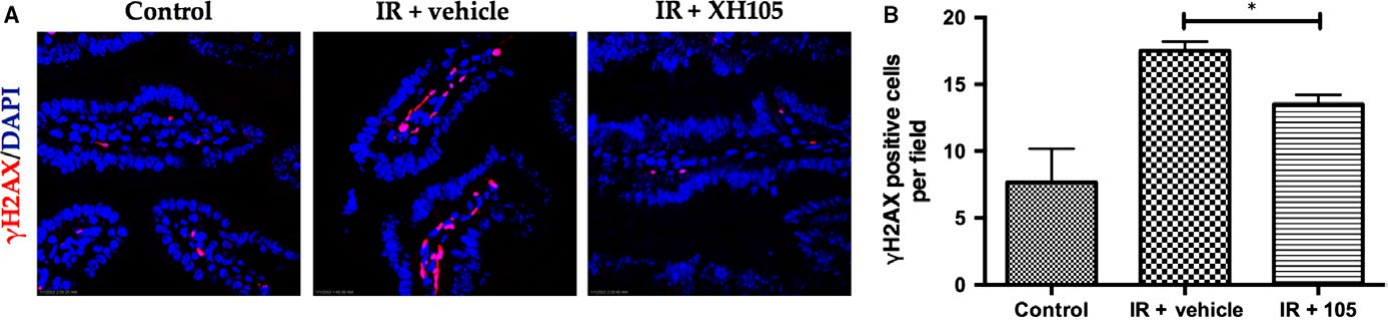
XH‐105 attenuates DNA damage of mice after 9.0 Gy total body irradiation (TBI). The small intestines of control mice, vehicle‐treated mice and XH‐105 treated mice were obtained at 3.5 d after 9.0 Gy TBI. A, Representative immunofluorescence images for the expression of γH2AX of the small intestines (red, γH2AX; blue, DAPI). B, Histogram demonstrating Quantitative analysis of γH2AX‐positive cells per view field. The results are represented as mean ± SEM, n = 5 mice per group. *P < 0.05. Scale bar: 10um

To determine whether XH‐105 treatment could reduce TBI‐induced DNA damage, histone H2AX phosphorylation was analysed. As demonstrated in Figure 6, there was an increase in γH2AX in intestinal sections from the IR group compared with the control group. XH‐105 treatment decreased H2AX phosphorylation in intestinal sections compared with vehicle‐treated mice after 9.0 Gy irradiation. The results indicated that XH‐105 could reduce IR‐induced DNA damage to the small intestine.

### 3.7 XH‐105 protects the small intestine against radiation‐induced injury at least in part via the p53 signalling pathway

**Figure 7:**
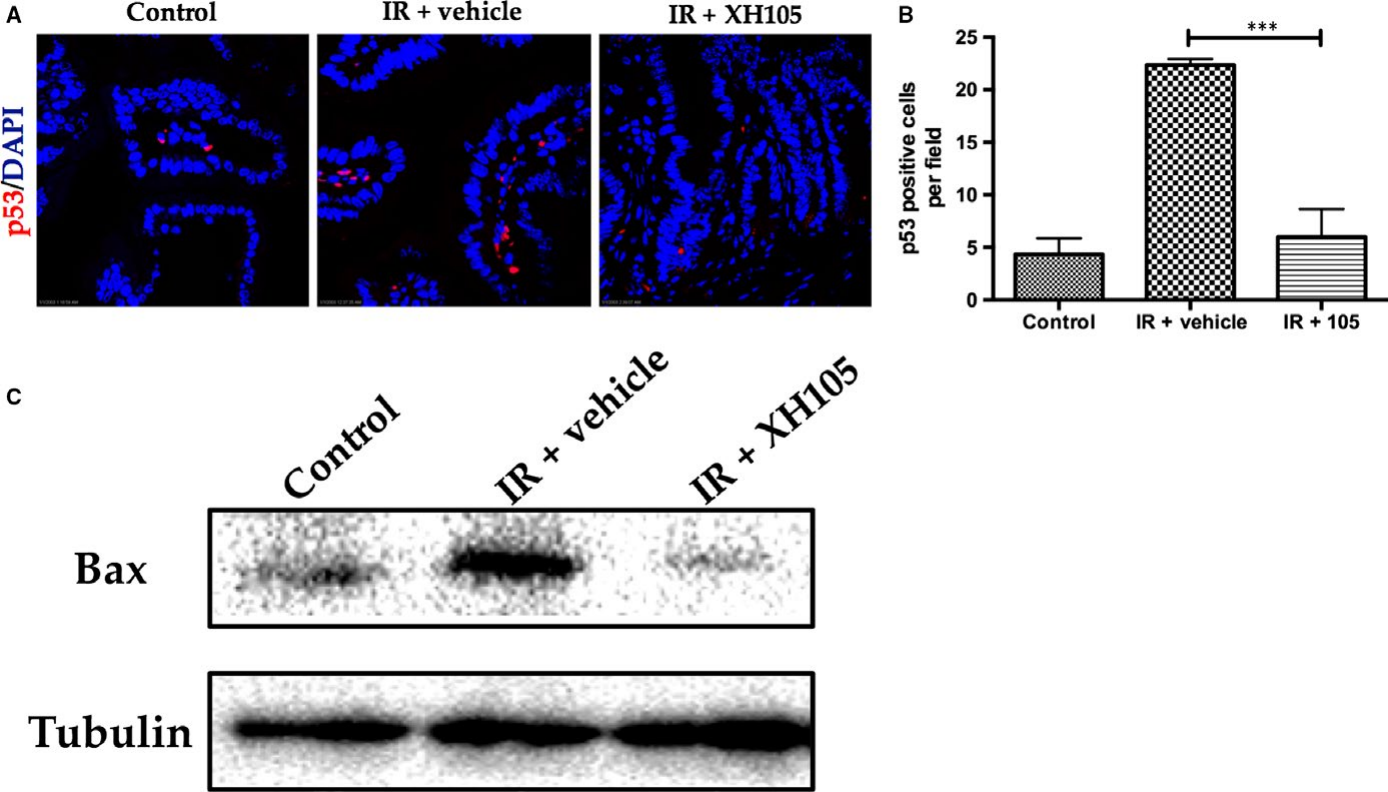
XH‐105 decreases the expression of p53 and Bax of the small intestine after 9.0 Gy total body irradiation (TBI). The small intestinal sections of control, IR + vehicle and IR + 105 mice were gained at 3.5 d after 9.0 Gy TBI. A, Representative immunofluorescence images for the expression of p53 of the small intestines (red, p53; blue, DAPI). B, Histogram showing Quantitative analysis of p53‐positive cells per field of view. C, Western blot for Bax and tubulin in the intestinal crypts from non‐IR mice, vehicle‐treated mice and XH‐105 treated mice at 3.5 d after 9.0 Gy TBI. The results are represented as mean ± SEM, n = 5 mice per group. ***P < 0.005. Scale bar: 10um

To investigate the mechanisms by which XH‐105 protects against radiation‐induced intestinal injuries, the expression of p53 was determined by immunofluorescence (Figure 7A). We isolated the intestinal crypt cells and evaluated the expression of *Bax* by Western blot at 3.5 days after 9.0 Gy TBI (Figure 7C). IR increased the expression of p53 in the small intestine compared with the control group. In contrast, mice treated with XH‐105 down‐regulated the expression of p53 (Figure 7B). Similarly, XH‐105 decreased the expression of Bax. Taken together, these findings suggested that XH‐105 protects the small intestine from IR at least by the p53 signalling pathway.

## 4 DISCUSSION

The gastrointestinal (GI) system is one of the most sensitive organs to radiation, and radiation‐induced intestinal injuries seriously affect the efficacy of tumour radiotherapy.^13^ Therefore, development of an effective method and drug to mitigate the radiation‐induced intestinal injuries needs to be explored. Many studies have reported that Chinese herbal medicines or extracts and other drugs may be able to reduce TBI‐induced injuries in the brain, oesophagus and haematopoietic system of irradiated animals,^14^, ^15^, ^16^, ^17^, ^18^ but the study of protective drugs in IR‐induced intestinal injuries still needs to be improved.^19^, ^20^, ^21^ In the present study, we observed that the new compound XH‐105 had protective effects on radiation‐induced intestinal injuries.

After receiving radiation, various degrees of villi blunting and fusion may occur; villous epithelial cell attenuation and hypertrophy and severe loss of crypts lead to the destruction of epithelial cell homeostasis and epithelial integrity.^22^ Intestinal epithelial cells cannot easily maintain intestinal absorption and defense functions.^23^ Our results demonstrated that the intestinal crypt‐villus structure in XH‐105‐treated mice was well preserved after 9.0 Gy TBI. The intestinal epithelium is one of the most rapidly self‐renewing organizations in mammals and is continuously renewed by intestinal epithelial stem cells located in the crypts.^24^, ^25^ Intestinal epithelial stem cells renewal is identified by expression of Lgr5.^26^, ^27^ Under physiological conditions, epithelial homeostasis is maintained by proliferative cells in crypts, and the small intestinal crypt cells are particularly sensitive to IR due to their high proliferative rate.^28^ The stem cell niche located at the bottom of intestinal crypts contains Paneth cells, which can produce lysozymes. Previous studies have shown that the number of Paneth cells decreases with the loss of Lgr5^+^ stem cells.^29^, ^30^ We found that the number of Lgr5^+^ intestinal stem cells increased in the XH‐105‐treated group after 9.0 Gy TBI and that the Lgr5^+^ intestinal stem cells differentiated into more Paneth cells and villus cells. Thus, XH‐105 may play a protective role against IR‐induced intestinal injuries by improving the proliferation and differentiation of Lgr5^+^ intestinal stem cells. The increased expression of Ki67, a proliferative marker in the small intestine, in the XH‐105‐treated mice indicated the recovery of the intestinal cells after IR‐induced injuries. These results indicated that XH‐105 may have a protective effect on irradiation‐induced intestinal injury.

Many studies have shown that IR‐induced tissue damage increases the amount of apoptotic cells.^31^, ^32^, ^33^ Caspases are a family of genes important for maintaining homeostasis by regulating apoptosis and inflammation.^34^ Caspases involved in apoptosis have been subclassified by their mechanism of action into initiator caspases (caspase‐8 and ‐9) and executioner caspases (caspase‐3, ‐6 and ‐7). We found that XH‐105 decreased the number of apoptotic cells in the small intestines by inhibiting the expression of caspase‐8 and caspase‐9.

Phosphorylated H2AX is a variant form of histone H2A, which has been widely used as a marker for DNA double‐strand breaks.^35^ In this study, we observed that the expression of γH2AX decreased in XH‐105‐treated mice after TBI compared with the vehicle‐treated mice. Radiation induces DNA damage directly through ROS^36^ and destroys the expression of proteins in cells,^37^ activating p53.^32^, ^33^, ^36^, ^38^, ^39^, ^40^ Radiation activates p53 in the GI epithelium, and p53‐mediated apoptosis has been implicated in regulating the intestinal injuries.^22^, ^41^ It is well known that p53 activates genes that regulate cell cycle checkpoints, DNA damage and repair and apoptosis.^42^ In addition, p53 can promote apoptosis through interactions with Bcl‐2 family proteins, such as Bax, in the cytoplasm.^41^, ^43^ Studies reported that *Bax^−/−^* and *Bak1^−/− ^*mice reduced the apoptosis of epithelial cells after exposure to irradiation.^44^, ^45^ Treatment with XH‐105 decreased the expression levels of Bax and p53. These data suggested that XH‐105 mitigated DNA damage induced by IR and radiation‐induced intestinal injuries via the p53‐dependent apoptosis signalling pathway.

Our studies show protective effects of XH‐105 against radiation‐induced intestinal injury, which may attenuate radiation‐induced intestinal damage via the p53 signalling pathway. XH‐105 is a promising novel compound to be used as a radioprotector, and the mechanism of radiation enteritis and therapy methods need to be further investigated.

## CONFLICT OF INTEREST

The authors confirm that there are no conflicts of interest.

## AUTHORS’ CONTRIBUTIONS

Deguan Li and Hongqi Tian conceived and designed the experiments. Hongqi Tian and Ying Cheng designed and synthesized the new compound XH‐105. Deguan Li, Yinping Dong, Qinlian Hou and Jing Wu carried out the follow‐up experiments, analysed the data, and interpreted the results. Deguan Li, Yinping Dong, Hongqi Tian and Ying Cheng contributed to the data analysis and the manuscript preparation. Wei Zhang contributed to the potential application of the compound XH‐105 and the manuscript revision.
