# Supplementary material for: The protective effects of XH‐105 against radiation‐induced intestinal injury
Source: J Cell Mol Med. 2019 Jan 20;23(3):2238–47. doi: 10.1111/jcmm.14159 (PMC6378229; doi:10.1111/jcmm.14159)
Supplement: Supplementary file 1 [file JCMM-23-2238-s001.docx]

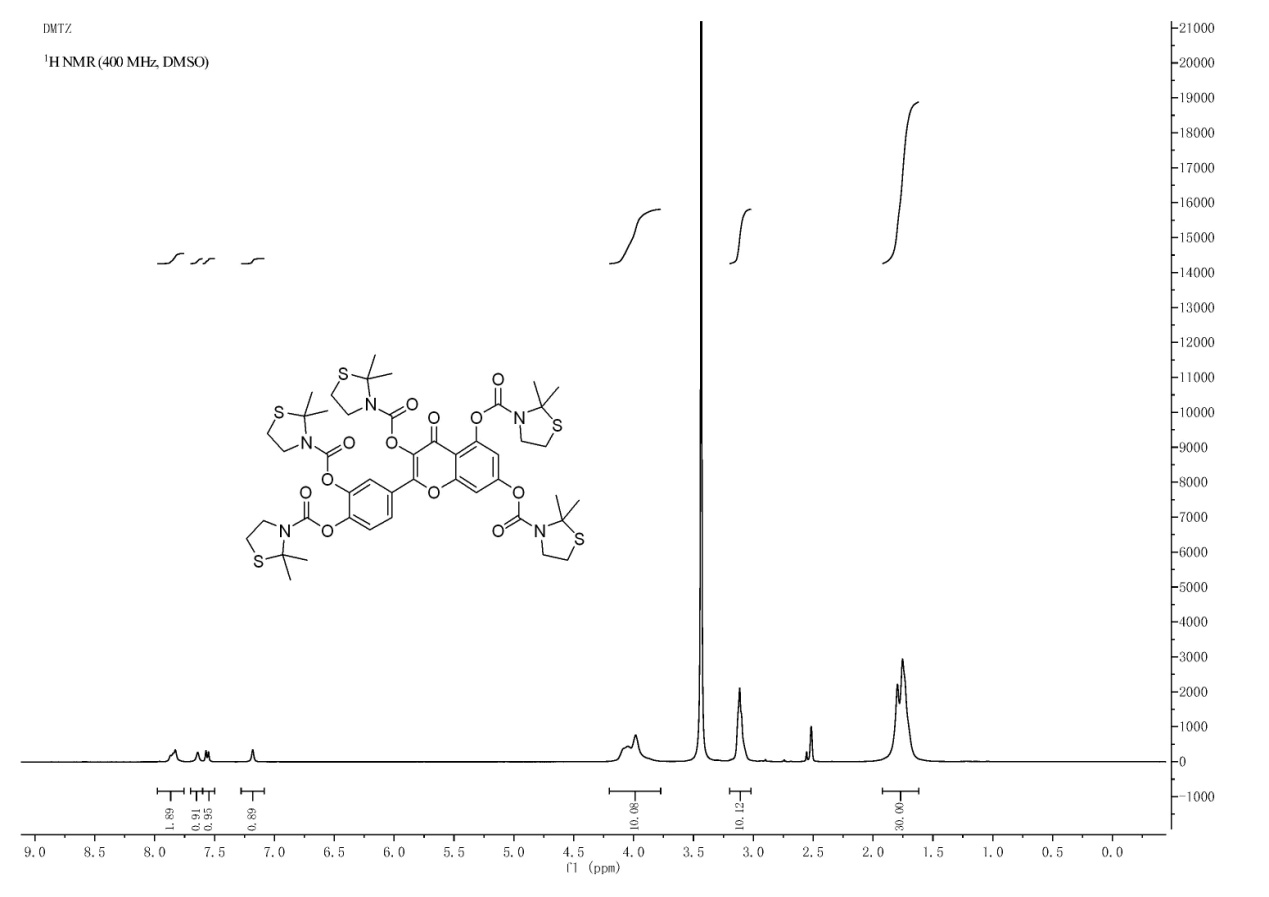


**Figure S1a. ^1^HNMR Spectra of DMTZ. DMTZ was characterized by ^1^HNMR (400MHz, d-DMSO).**


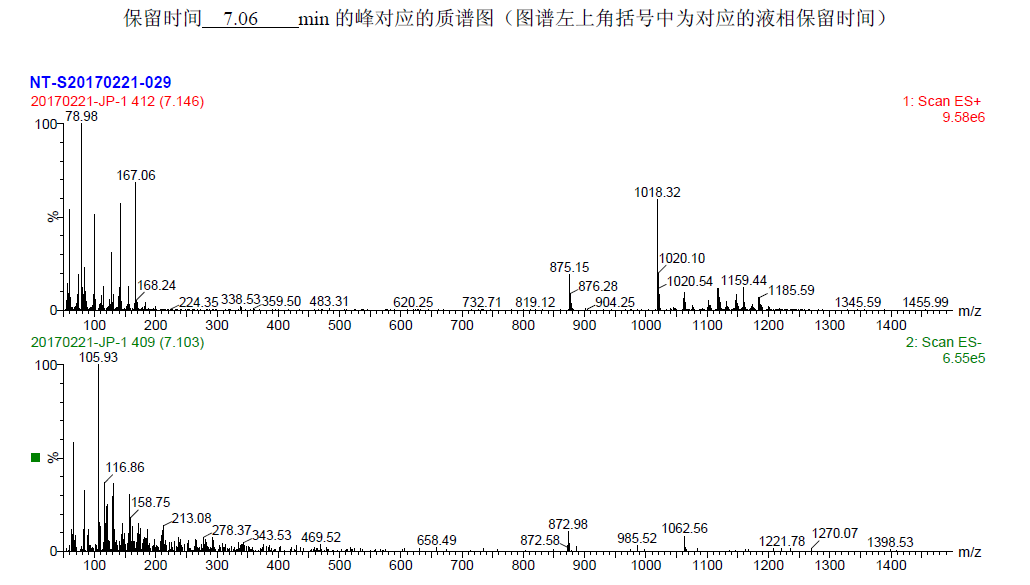


**Figure S1b. LC-MS Spectra of DMTZ. DMTZ was identified by LC-MS spectra to find the target [M+H]^＋^=1018.32.**
